# The role of hyaluronic acid and hyaluronidase-1 in obstructive sleep apnoea

**DOI:** 10.1038/s41598-020-74769-4

**Published:** 2020-11-10

**Authors:** Martina Meszaros, Adrian Kis, Laszlo Kunos, Adam Domonkos Tarnoki, David Laszlo Tarnoki, Zsofia Lazar, Andras Bikov

**Affiliations:** 1 Department of Pulmonology, Semmelweis University, Budapest, 1083 Hungary; 2 Medical Imaging Centre, Semmelweis University, Budapest, 1082 Hungary; 3 Manchester University NHS Foundation Trust, Manchester, M13 9WL UK; 4 Division of Infection, Immunity and Respiratory Medicine, University of Manchester, Manchester, M13 9NT UK

**Keywords:** Medical research, Biomarkers

## Abstract

Biological functions of hyaluronic acid (HA) depend on its molecular size. High-molecular weight HA (HMW-HA) is an important component of the endothelial wall and has anti-inflammatory and antioxidant properties. Under inflammation or hypoxia, HMW-HA is degraded by hyaluronidases, such as HYAL-1 resulting in pro-inflammatory low-molecular weight fragments. Obstructive sleep apnoea (OSA) is characterised by intermittent hypoxia and systemic inflammation. Our aim was to evaluate circulating HMW-HA and HYAL-1 in OSA. We recruited 68 patients with OSA and 40 control volunteers. After full-night sleep study blood samples were taken for HMW-HA and HYAL-1 measurements. HYAL-1 levels were significantly higher in patients with OSA compared to controls (0.59/0.31–0.88/ng/mL vs. 0.31/0.31–0.58/ng/mL; p = 0.005) after adjustment for gender, age, BMI and smoking. There was a trend for reduced HMW-HA concentrations in OSA (31.63/18.11–59.25/ng/mL vs. 46.83/25.41–89.95/ng/mL; p = 0.068). Significant correlation was detected between circulating HMW-HA and apnoea-hypopnoea-index (r = − 0.195, p = 0.043), HYAL-1 and apnoea-hypopnoea-index (r = 0.30, p < 0.01) as well as oxygen desaturation index (r = 0.26, p < 0.01). Our results suggest that chronic hypoxia is associated with increased plasma HYAL-1 concentration and accelerated HMW-HA degradation. Altered hyaluronan metabolism may be involved in the inflammatory cascade potentially leading to endothelial dysfunction in OSA.

## Introduction

Obstructive sleep apnoea (OSA) is the most common sleep-related breathing disorder which is characterised by the repetitive collapse of the upper airways during sleep resulting in chronic intermittent hypoxaemia (CIH) and frequent arousals with sleep fragmentation. These factors lead to enhanced oxidative stress and consequential systemic inflammation which are associated with altered concentrations of circulating pro-inflammatory^1,2^ and anti-inflammatory^3,4^ biomarkers. Understanding the role of inflammation in OSA is of importance as it could be a link to the development of cardiometabolic comorbidities.

Hyaluronic acid (HA) is a glycosaminoglycan which is an essential constituent of extracellular matrix (ECM) in several tissues, such as tracheobronchial mucosa and the endothelium^5^. The biological functions of HA are related to its molecular size. Under physiological conditions extracellular high molecular weight HA (HMW-HA; > 1000 kDa) plays a role in structural functions of ECM, tissue regeneration and morphogenesis, by interacting with several types of HA-binding proteins. Under inflammatory conditions HMW-HA acts as an important anti-inflammatory and anti-angiogenic molecule ^6^. In contrast, low molecular weight HA (LMW-HA; 150–350 kDa) displays pro-inflammatory properties, such as stimulating macrophage activation and increasing the expression of cytokines and growth factors^7^. HMW-HA is degraded by two different mechanisms, predominantly via hyaluronidases (HYAL) and in lesser extent via oxidative stress. Out of the six hyaluronidases, HYAL-1 and HYAL-2 are responsible for the majority of LMW-HA production in the somatic tissues^8^. HYAL-2, as a cell surface protein that starts the cleavage of HMW-HA and HYAL-1 further digests them in liposomes^9^. Moreover, HYAL-1 can be secreted by the cells, it is measurable in the circulation and can be internalised via endocytosis by endothelial cells^10^. Reactive oxygen species (ROS) and reactive nitrogen species (RNS) can degrade HMW-HA by direct depolymerisation^11^.

Clinical data provided evidence that inflammation is present in the upper and lower airways in OSA which potentially contribute to systemic inflammation^12^. Hyaluronan metabolism has an important role in lung pathophysiology during inflammation. Altered HA production has been detected in airway diseases characterised by inflammation and oxidative stress, such as asthma and COPD^13,14^. Fragments derived from HMW-HA stimulate several inflammatory cells in the airways, such as alveolar macrophages to produce mediators and these mechanisms enhance inflammation^7^. The expression of HYAL enzymes is highly induced in the airway epithelial cells by pro-inflammatory cytokines suggesting that the epithelium could be the source for LMW-HA in airway inflammation^13^. Preliminary reports detected elevated HYAL-1 activity in the airways of asthma and COPD patients^15^. Moreover, increased enzymatic degradation of HMW-HA in bronchoalveolar lavage fluid and serum of COPD patients were associated with airflow limitation^14,16^. The same mechanisms can contribute to accelerated metabolism of HMW-HA and enhanced airway and systemic inflammation in OSA. However, HA levels have not been analysed in this disease. Therefore, our aim was to investigate the levels of HMW-HA and HYAL-1 in patients with OSA and understand the role of hyaluronan metabolism in the pathogenesis of OSA.

## Results

### Patient characteristics

Sixty-eight patients were diagnosed with OSA (median age: 53 years/47–64/, 49 men). Twenty-five of them had mild (AHI 5–14.9 events/h), 16 moderate (AHI 15–29.9 events/h) and 27 severe (AHI > 30 events/h) disorder. Subjects’ characteristics and comparisons between the OSA and control groups are shown in Table 1. Patients with OSA were older (p = 0.08) and had significantly higher BMI, systolic (SBP) and diastolic blood pressure (DBP) and ESS values. They had higher levels of CRP, glucose, total triglyceride and lipoprotein (a) and lower levels of HDL-C (all p < 0.01). There was no difference in total cholesterol and LDL-C levels (p = 0.43; p = 0.73). No differences were detected in the prevalence of comorbidities except for hypertension (p < 0.01). The OSA group had higher AHI, ODI, SPT, TST, and TST90% and lower MinSatO_2_ values (all p < 0.05).

**Table 1:** Subjects’ characteristics.

	Control (n = 40)	OSA (n = 68)	Total (n = 108)	p
Age (years)	51/39–60/	53/47–64/	53/44–62/	0.08
Males (%)	28	72	56	< 0.01
BMI (kg/m^2^)	24.75 ± 4.48	31.98 ± 5.97	29.30 ± 6.48	< 0.01
Hypertension (%)	38	71	58.3	< 0.01
Diabetes (%)	13	21	17.6	0.29
Dyslipidaemia (%)	30	37	34.3	0.47
Cardiovascular disease (%)	10	15	13	0.48
Chronic cardiac failure (%)	5	12	9	0.24
Cardiac arrythmia (%)	8	21	15.7	0.07
Asthma (%)	10	15	13	0.48
COPD (%)	5	9	7	0.46
Smokers (%)	5	46	30.6	< 0.01
Pack years	0/0–0/	0/0–10/	0/0–5/	< 0.01
SBP (mmHg)	120/110–130/	132/124–138/	130/120–136/	< 0.01
DBP (mmHg)	70/70–80/	80/74–89/	78/70–84/	< 0.01
CRP (mg/l)	1.47/0.83–2.83/	3.51/1.76–8.27/	2.175/1.15–4.85/	< 0.01
Glucose (mmol/l)	4.6/4.2–5.2/	5.3/4.9–6.7/	5.1/4.7–6.1/	< 0.01
Cholesterol (mmol/l)	5.64 ± 1.1	5.45 ± 1.26	5.52 ± 1.20	0.43
HDL-C (mmol/l)	1.61/1.39–1.95/	1.21/0.98–1.33/	1.33/1.14–1.63/	< 0.01
LDL-C (mmol/l)	3.39 ± 0.98	3.46 ± 1.05	3.44 ± 1.02	0.73
Triglyceride (mmol/l)	1.13/0.88–1.39/	1.67/1.25–2.17/	1.35/1.07–1.96/	< 0.01
Lipoprotein (a) (mmol/l)	0.25/0.07–0.56/	0.74/0.37–0.99/	0.32/0.12–0.67/	< 0.01
AHI (1/h)	1.90/0.78–2.70/	18.1/10.3–41.5/	9.25/2.38–28.23/	< 0.01
ODI (1/h)	0.75/0.20–1.53/	15.9/8.5–33.7/	6.95/1.08–22.38/	< 0.01
AI (1/h)*	45.11 ± 18.85	48.06 ± 14.57	46.09 ± 17.36	0.65
SPT (min)*	422.5/398.3–439.3/	451.0/404–485/	435.0/403.6–479.1/	0.04
TST (min)*	381.26 ± 45.93	415.32 ± 62.62	402.27 ± 58.81	0.03
TST90% (%)	0.0/0.0–0.8/	3.45/0.53–12.35/	0.7/0.0–6.9/	< 0.01
MinSatO_2_ (%)	91/89–93/	83/78–87/	87/81–91/	< 0.01
ESS	5.5/2.0–6.8/	6/4–9/	6/4–8/	0.02

Data are presented as mean ± standard deviation or median/25–75% percentile/.

*AHI* apnoea-hypopnoea index, *AI* arousal index, *BMI* body mass index, *COPD* chronic obstructive pulmonary disease, *CRP* C-reactive protein, *DBP* diastolic blood pressure, *ESS* Epworth Sleepiness Scale, *HDL-C* high-density lipoprotein cholesterol, *LDL-C* low-density lipoprotein cholesterol, *MinSatO*_*2*_ minimal oxygen saturation, *ODI* oxygen desaturation index, *SBP* systolic blood pressure, *SPT* sleep period time, *TST* total sleep time, *TST90% *total sleep time spent with oxygen saturation below 90%.

*Data available in 23 control subjects and 37 patients with OSA.

### Circulating HA and HYAL-1 levels

**Figure 1:**
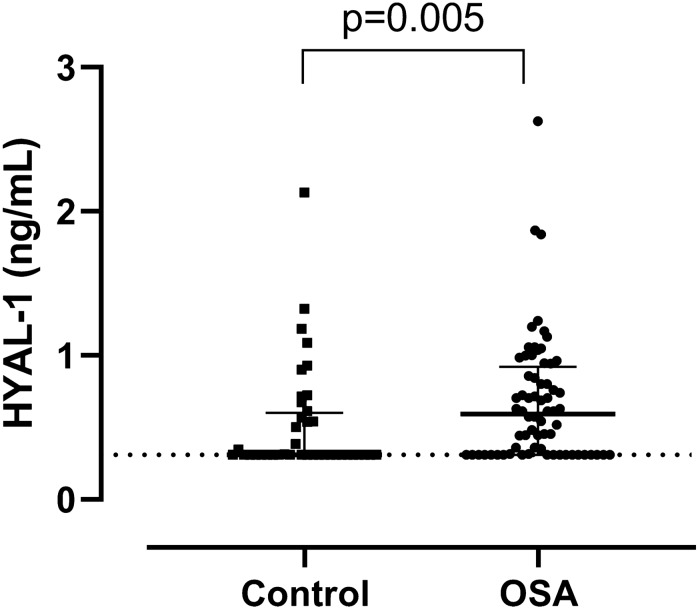
HYAL-1 (hyaluronidase-1) concentrations between the control and OSA (obstructive sleep apnoea) groups. Data are presented as median with interquartile range. Dashed line means the limit of detection.

**Figure 2:**
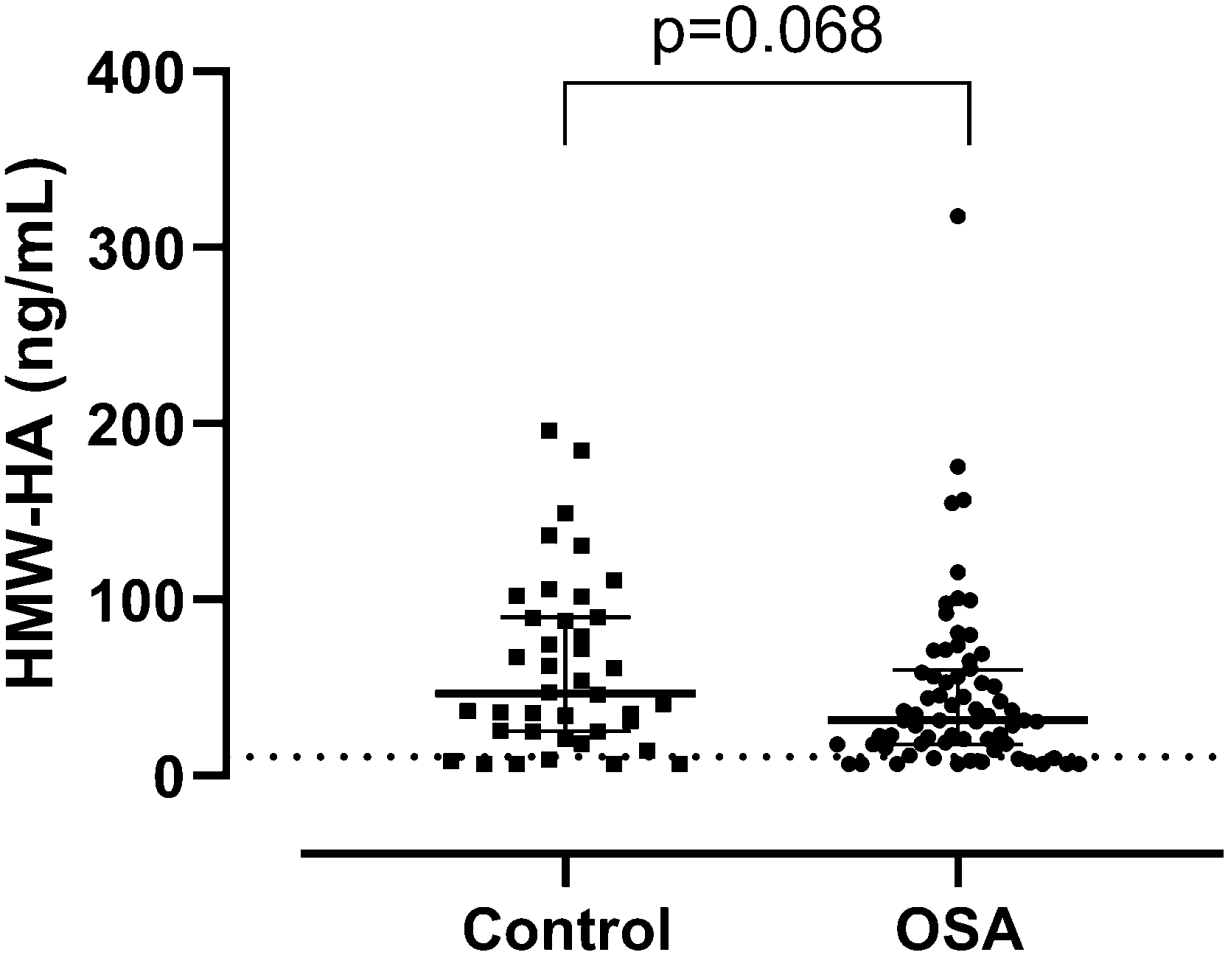
HMW-HA (high molecular weight hyaluronic acid) concentrations between the control and OSA (obstructive sleep apnoea) groups. Data are presented as median with interquartile range. Dashed line means the limit of detection.

Plasma HYAL-1 concentrations were significantly higher (0.59/0.31–0.88/ng/mL vs. 0.31/0.31–0.58/ng/mL; p = 0.005, Fig. 1) and HMW-HA levels were lower (31.63/18.11–59.25/ng/mL vs. 46.83/25.41–89.95/ng/mL; p = 0.068, Fig. 2) in the OSA group compared to the controls after adjustment for age, gender, BMI and smoking. There was a significant difference between the OSA and control groups for HMW-HA/HYAL-1 ratio (61.8/25.74–107.54/ vs 118.12/44.23–260.39; p = 0.005). After adjustment for hypertension and cardiovascular diseases HYAL-1 concentrations remained significantly higher (p = 0.005), HMW-HA levels were lower (p = 0.068) in OSA group. The difference between the two groups remained significant for HMW-HA/HYAL-1 ratio (p = 0.005).

Concentrations below the detection limit were measured in 19% (n = 13) of OSA group and in 15% (n = 6) of control group for HMW-HA (p = 0.59), and in 28% (n = 19) of patients with OSA and in 58% (n = 23) of control patients for of HYAL-1 (p = 0.002).

We noticed 10 outliers (2 controls and 4 patients for HMW-HA as well as 1 control and 3 patients for HYAL-1). These subjects were not different in their general characteristics or comorbidities from the others. After excluding them, the HYAL-1 levels and HMW-HA/HYAL-1 ratio were still significantly different (p = 0.013; p = 0.002) and the difference in HMW-HA concentrations between the two groups became significant (p = 0.027).

When excluding patients with chronic airway disease and chronic heart failure (33 controls vs. 50 patients) we significantly lower HMW-HA (28.62/16.50–44.86/ vs. 47.45/25.38–89.90/ng/ml, p = 0.021) and significantly higher HYAL-1 (0.61/0.31–0.94/ vs. 0.31/0.31–0.54/ng/ml, p = 0.002) levels were detected in OSA. The difference in HMW-HA/HYAL-1 was also significant (p = 0.001).

Eighteen participants were treated with statins and three patients were treated with systemic steroids (low dose methylprednisolone). After adjustment for the statin and steroid usage, HYAL-1 levels (p = 0.005) and the HMW-HA/HYAL-1 ratio (p = 0.005) were still significantly different and the difference in HMW-HA concentrations between the two groups did not change (p = 0.068).

### Relationship between HMW-HA and HYAL-1 levels and clinical variables

**Figure 3:**
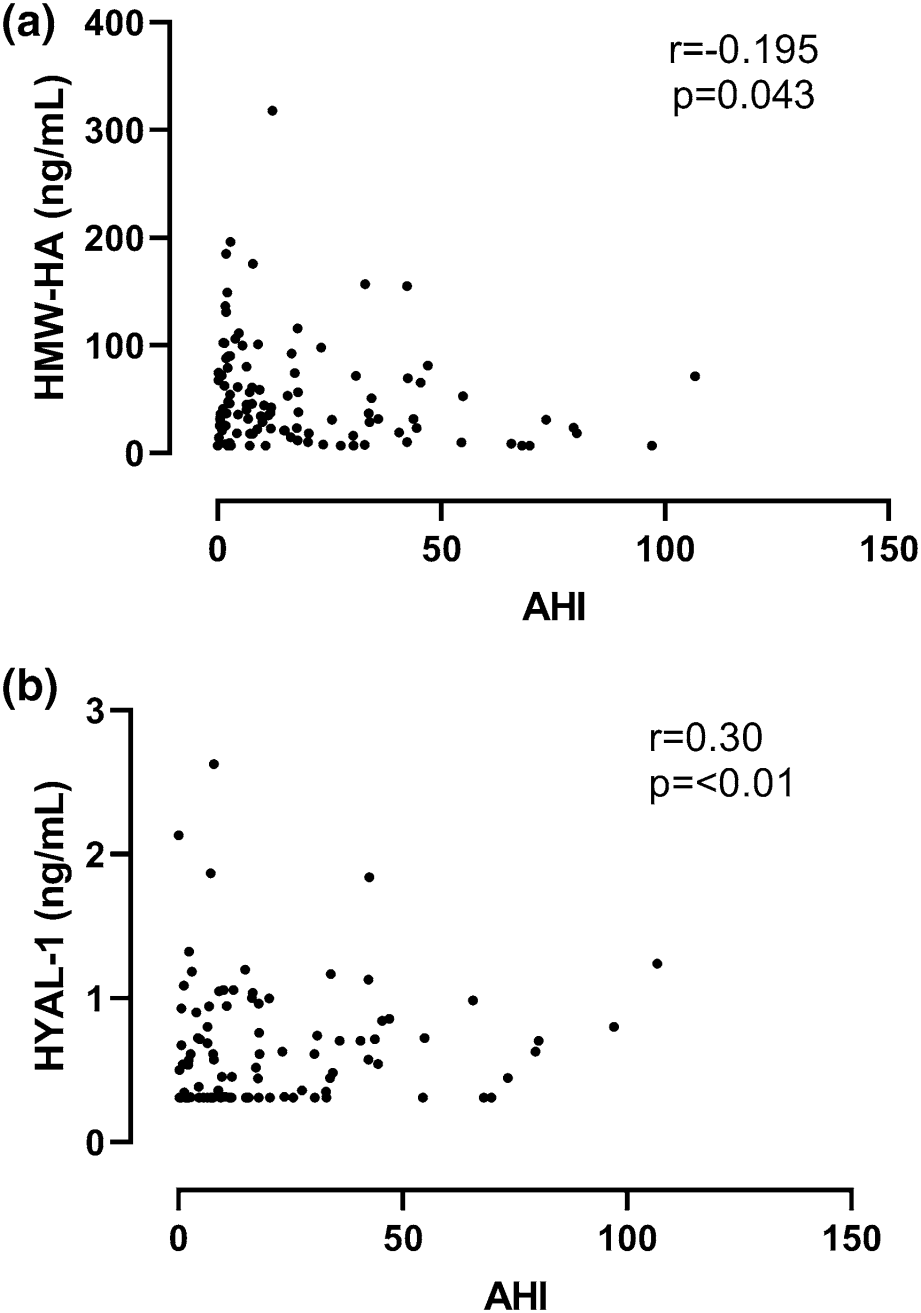
**(a)** Correlation between HMW-HA (high molecular weight hyaluronic acid levels and AHI (apnoea-hypopnoea index). **(b)** Correlation between HYAL-1 (hyaluronidase-1) levels and AHI (apnoea-hypopnoea index).

**Figure 4:**
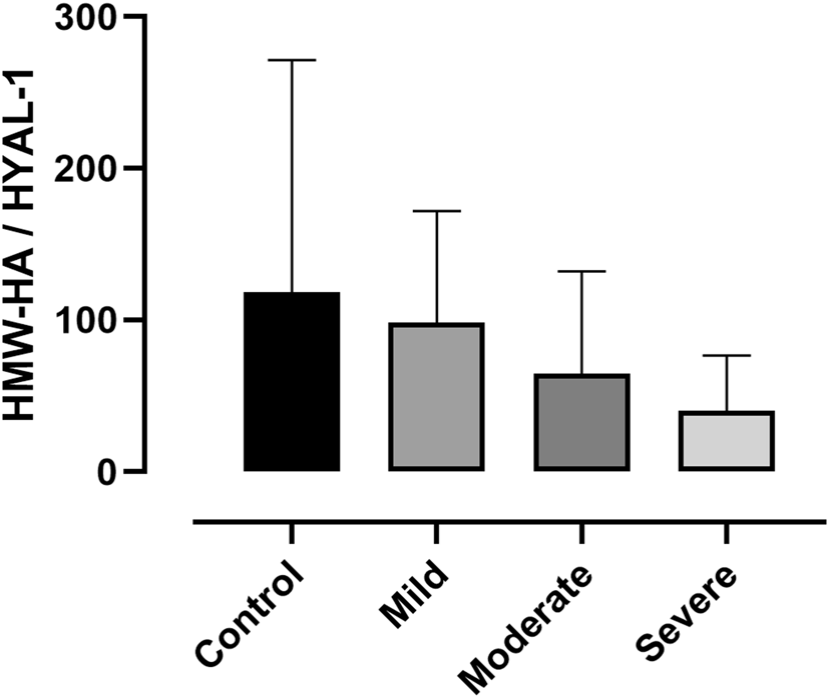
HMW-HA/HYAL-1 ratio along groups of increasing disease severity. Data are presented as median with interquartile range.

We observed a significant negative correlation between plasma HMW-HA and AHI (r = − 0.195, p = 0.043, Fig. 3a). However, there was no correlation between HMW-HA and the other sleep parameters (all p > 0.05). HYAL-1 levels significantly correlated with AHI (r = 0.30, p < 0.01, Fig. 3b), ODI (r = 0.26, p < 0.01) and correlated with TST90% (r = 0.186, p = 0.057) and MinSatO_2_ (r = − 0.184, p = 0.059), however the latter two correlations did not reach the level of significance. When comparing biomarker levels along subgroups of increasing OSA severity, there was a significant difference in HYAL-1 concentration (p = 0.03) as well as HMW-HA/HYAL-1 ratio (p = 0.005, Fig. 4), but not in HMW-HA levels (p = 0.12). We found a direct correlation between HMW-HA levels and age (r = 0.41, p < 0.01). HYAL-1 concentrations were higher in men than women (0.56/0.31–0.85/ng/mL vs. 0.31/0.31–0.72/ng/mL, p = 0.01), directly related to glucose (r = 0.32; 0.002), CRP (r = 0.30; 0.005) and triglyceride levels (r = 0.24; 0.014) and there was an indirect correlation with HDL-C concentrations (r = − 0.21; 0.036). None of the other correlations between the clinical variables as well as HMW-HA or HYAL-1 were significant (all p > 0.05). HA levels did not correlate with HYAL-1 levels (r = − 0.12; p = 0.20). No correlation was detected between HA and HYAL-1 levels in either control subjects (r = − 0.25; p = 0.12) or patients with OSA (r = 0.06; p = 0.63).

### The effect of obesity on HMW-HA and HYAL-1 levels

There was a significant correlation between HYAL-1 and BMI in the total cohort of subjects (r = 0.25; p = 0.01); however, this correlation became insignificant when controls (r = 0.25; p = 0.12) or patients with OSA (r = 0.06; p = 0.65) were investigated separately. No correlation was observed between HMW-HA and BMI either in all subjects, or in the control or OSA subgroups separately (all p > 0.05). We compared HMW-HA and HYAL-1 levels between non-obese and obese (BMI ≥ 30 kg/m^2^) controls and patients with OSA after adjustment for age, gender, smoking and AHI. There was no difference in HYAL-1 concentrations between the non-obese (N = 25; 0.61/0.36–0.86/ng/mL) and obese (N = 43; 0.54/0.31–0.89/ng/mL) patients with OSA (p = 0.68). Interestingly, HYAL-1 levels were significantly higher in obese controls (N = 6; 0.72/0.59–0.99/ng/mL) compared to the non-obese subjects (N = 34; 0.31/0.31–0.47/ng/mL) (p = 0.012). The levels of HMW-HA did not differ between non-obese and obese participants either in controls (p = 0.87; 46.83/25.48–90.04/ng/mL vs 56.53/23.98–77.24/ng/mL), or in OSA group (p = 0.63; 34.42/22.22–50.90/ng/mL vs 30.95/12.34–67.49/ng/mL).

## Discussion

We reported higher plasma HYAL-1 and lower HMW-HA levels in OSA. Chronic intermittent hypoxia, enhanced oxidative stress and inflammation characterising OSA may explain our results.

Chronic intermittent hypoxia influences the expression of several hypoxia-related transcriptional factors, such as hypoxia-inducible factor-1 and nuclear factor kappa-B (NF-kB) which play role in inflammatory responses^17,18^. Gao et al. have demonstrated that hyaluronan metabolism is regulated by hypoxia in vitro as it upregulates the expression of HYAL^19^. In line with this, plasma HYAL-1 levels were associated with the markers of overnight hypoxia in our current study. Although CIH can upregulate the anti-inflammatory HMW-HA, it is quickly degraded by increased concentrations of HYAL enzymes resulting higher LMW-HA levels^19^. Hypoxia can also increase the expression of CD44 which is the most important receptor of HA fragments and it is expressed on a wide range of inflammatory cells^20^. This may result in a consequentially higher HA uptake from the circulation.

Increased production of ROS and RNS is well described in OSA^21^. Oxidative stress can degrade HA through direct and indirect mechanisms. HA polymers are susceptible to direct oxidation by ROS and RNS^11^. Oxidative stress can also induce the expression of HYAL^22^. In OSA patients CIH decreases the plasma concentration of superoxide dismutase (SOD)^23^. In SOD3 deficient mice elevated lung HYAL expression was detected due to hypoxic injury. Moreover, the loss of SOD3 increased the hypoxia-induced HYAL expression and activity in human pulmonary artery smooth muscle cells in vitro^24^.

Pro-inflammatory cytokines, such as interleukin 1-beta (IL-1β) and tumour necrosis factor-alpha (TNF-α) increase HA levels by upregulating HA-synthase enzymes^25^. However, these cytokines elevate HYAL levels in plasma as well as in the endothelium leading to an accelerated HA clearance from the circulation. Previous studies have shown that LMW-HA fragments stimulate the production of IL-1β, IL-8 via NF-kB signalling^26^ and potently enhance the activation and recruitment of neutrophils^27^. The activation of NF-kB and consequential elevated levels of these cytokines are well described in OSA^17^.

In contrast to the pro-inflammatory molecules, the role of anti-inflammatory processes in OSA has not been fully elucidated. Only a few anti-inflammatory biomarkers have been examined with altered levels in OSA^3,4^. HMW-HA is a potent anti-inflammatory and immunosuppressive molecule. It is well known that HMW-HA was able to down-regulate the genes of NF-kB and several interleukins^6^. Moreover, HMW-HA is capable of neutralizing intra- and extracellular ROS^28^. Protective activation of HYAL enzymes has been also demonstrated by decreasing the expression of TNF-α and IL-6^29^. A recent study has shown that HYAL suppressed neutrophil recruitment and cytokine production in a murine model of acute inflammation^30^. However, the immunoregulatory functions of HYAL-1 are contradictory. High activity of HYAL enzymes elevate pro-inflammatory LMW-HA levels and itself can damage endothelial glycocalyx resulting in endothelial dysfunction and vascular inflammation^31^.

OSA is associated with increased cardiovascular morbidity and mortality^32^. Alterations of hyaluronan metabolism have been demonstrated in association with endothelial dysfunction through glycocalyx destabilization with attenuated endothelial nitric oxide availability contributing to atherosclerosis^31,33^. The degradation of HMW-HA under inflammatory conditions reduces extracellular HA viscosity and increase tissue permeability^34^ resulting extravasation of macrophages to the arterial wall. The damaged endothelium facilitates the adhesion of leukocytes and platelets via adhesion molecules, and the levels were found to be elevated in OSA^2,35^. Dogné et al. demonstrated that the lack of HYAL-1 is associated with lower levels of P-selectin suggesting that HYAL-1 deficiency may be protective in endothelial dysfunction^33^. Elevated HYAL levels are implicated in intima aging^36^ and increased HYAL activity was detected in patients with coronary artery disease^37^ indicating its potential role in atherosclerosis. In addition, HYAL enzymes impair the endothelial mechanosensitive response by glycocalyx shedding and potentiate the dysregulation of vascular tone^38,39^. Supporting this, arterial stiffness was significantly associated with increased plasma HYAL concentrations suggesting that altered hyaluronan metabolism may be involved in the pathophysiology of hypertension^40^.

Obstructive sleep apnoea is often associated with obesity. Fat tissue is a major source for inflammatory molecules and obesity itself can be associated with elevated levels of inflammatory molecules^41^. The hyaluronic acid has a well-described role in adipogenesis^42^. We found a significant relationship between HYAL-1 levels and BMI, and HYAL-1 concentrations were higher in the obese compared to the non-obese controls. This suggests that obesity could partially contribute to our findings. Interestingly, plasma HMW-HA levels were not related to obesity. Most importantly, our primary analyses were adjusted to age, gender, smoking and BMI and the investigated molecules were not different in obese vs. non-obese OSA. This suggests that OSA-related changes in HMW-HA and HYAL-1 levels were not exclusively due to differences in BMI between the two groups.

Hyaluronic acid has been implicated in the pathophysiology on chronic airway diseases^13–15^ and chronic heart failure^43^. Therefore, we analysed our data also following exclusion of patients with these diseases. Exclusion did not affect the HYAL-1 or HMW-HA/HYAL-1 results, while the difference in HMW-HA concentrations between patients with OSA and controls became significant. This suggests that further studies analysing hyaluronic acid metabolism should take into account these diseases as confounders to their results.

This study has limitations. We did not measure LMW-HA as it less stable, has significantly shorter half-life compared to HMW-HA^44^ and is also quickly eliminated by the kidney^45^. However, to have a deeper understanding of hyaluronic acid metabolism in OSA, further studies may consider measuring LMW-HA as well. The levels of HYAL-1 and HMW-HA were below the detection limit in 39% and 18% of all cases. There are multiple techniques how to handle results with values below the lower limit of detection, including exclusion of these results, assigning the detection limit, detection limit value/√2 or zero. However, all these methods have limitations and replacing the values with the detection limit has the least bias^46,47^. Of note, this led to skewing our data. Therefore, our results need to be interpreted with caution. Nevertheless, significantly more patients had detectable levels of HYAL-1 than controls strengthening our conclusion that this enzyme is elevated in OSA. The study was powered to find differences in either HMW-HA or HYAL-1 between OSA and controls. The comparison has been adjusted to age, gender and BMI. As confirmed in this study, these parameters are often different in OSA compared to controls. Apart from the correlations with the BMI, we observed significant relationships between HMW-HA and age as well as HYAL-1 and gender. Although the primary analyses were adjusted on these factors, further studies should aim for better balanced case–control groups. The relationships between HYAL-1 and glucose, triglyceride as well as HDL-C levels also need to be interpreted carefully, as they may have been affected by OSA itself and does not necessarily represent and independent association. Circulating markers of hyaluronan metabolism were examined in diabetic patients and they were correlated with serum glucose and lipid parameters^48^. One may argue if these were independent associations as the potential presence of OSA was not considered. Nevertheless, the relationships between hyaluronan metabolism and metabolic profile should be investigated in non-OSA populations. Most notably, the correlation analyses in our study are exploratory and need to be interpreted with caution. Only 60 subjects had polysomnography, therefore data on TST, SPT and AI has to be interpreted carefully. Potentially, sleep fragmentation could also have contributed to our results, however this cannot be concluded as PSG has not been performed in everybody. In addition, being a more sensitive test, higher AHI values may have been obtained with PSG compared to PG as hypopnoeas resulting arousals, but no desaturation can be scored only with polysomnography. Therefore, correlations between biomarkers and AHI may have been stronger if only one sleep test has been used. None of the volunteers has been treated for their OSA before and a follow-up measurement after CPAP therapy in moderate-to-severe patients would have given additional information on the role of this molecular pathway. We believe that our study would provide an essential basis for sample size estimation when designing a prospective study evaluating the effect of CPAP.

In conclusion, our study indicates that plasma HYAL-1 levels are elevated in OSA and may decrease the circulating HMW-HA concentrations. These alterations potentially contribute to the enhanced systemic inflammation in OSA and could represent a potential link between OSA and its comorbidities.

## Methods

### Study design and subjects

We recruited 108 participants who were referred to the Sleep Unit of the Department of Pulmonology, Semmelweis University due to suspected OSA (i.e. symptoms of snoring, witnessed apnoea, daytime sleepiness, obesity or cardiometabolic comorbidities). None of the patients had previously been diagnosed with OSA and they had not been treated with continuous positive airway pressure (CPAP) or mandibular advancement devices (MAD). Patients with malignancy within 10 years, infection within 2 months, autoimmune disorders or uncontrolled chronic disease (for example acute heart failure, uncontrolled diabetes), were excluded.

In the evening, detailed medical history was taken, and patients filled out the Epworth Sleepiness Scale (ESS). Full-night, inpatient diagnostic cardiorespiratory polygraphy (PG, n = 48) or polysomnography (PSG, n = 60) were performed. The decision which diagnostic test to use was made by an expert somnologist who triaged the patients into low and high-likelihood groups. In the following morning between 6:00 and 8:00 a.m, systolic and diastolic blood pressure was measured and venous blood was taken to evaluate plasma HMW-HA and HYAL-1, as well as fasting serum glucose, total cholesterol, high-density lipoprotein cholesterol (HDL-C), low-density lipoprotein cholesterol (LDL-C), triglyceride, lipoprotein(a) and C-reactive protein (CRP) levels. Comorbidities were defined according to the patients’ report, medical history, and current medications. In particular, cardiovascular diseases included current or previous stable and unstable angina, stroke, transient ischaemic attack and significant atherosclerosis. Smoking status was defined as self-reported current smoking or previous history of smoking.

All procedures performed in studies involving human participants were in accordance with the ethical standards of the institutional and/or national research committee and with the 1964 Helsinki declaration and its later amendments or comparable ethical standards. The study protocol has been approved by the Semmelweis University Scientific Research Ethics Committee (TUKEB 30/2014, RKEB 172/2018) and all research was performed in accordance with relevant regulations. Written informed consent was provided by each volunteer.

### Sleep studies

Full-night cardiorespiratory polygraphy and polysomnography were performed using the Somnoscreen Plus Tele PSG (Somnomedics GmbH Germany) as described previously^49^. Sleep stages, movements and cardiopulmonary events were scored manually according to the American Academy of Sleep Medicine (AASM) guideline^50^. Apnoea was defined as a 90% decrease in airflow lasting for at least 10 s. Hypopnoea was defined as at least 30% decrease in airflow which lasted for more than 10 s with a ≥ 3% oxygen desaturation or an arousal. Total sleep time (TST), sleep period time (SPT) and minimal oxygen saturation (MinSatO_2_) were recorded, apnoea-hypopnoea index (AHI), oxygen desaturation index (ODI), total sleep time with saturation below 90% (TST90%) and arousal index (AI) were calculated to evaluate the severity of OSA. OSA was defined with an AHI > 5/h.

### Biomarker measurements

Blood samples were taken into EDTA tubes. They were processed within 30 min after collection and centrifuged at 4 °C for 10 min at 1500 rpm. Immediately after centrifugation plasma samples were separated and stored at − 80 °C until further analysis. Plasma HMW-HA and HYAL-1 levels were measured using commercially available ELISA kits (Human Hyaluronic Acid (HA) ELISA kit from Corgenix Inc (Catalogue number: 029-001), Colorado, USA; Human Hyaluronidase-1 (HYAL-1) ELISA Kit from Cusabio Technology Llc (Catalogue number: CSB-EL010918HU), Houston, USA). Plasma HMW-HA and HYAL-1 measurements were performed in duplicates according to the manufacturers’ instructions and the mean concentrations were used as inputs for analysis. The detection limits (DL) were 11 ng/mL for HMW-HA and 0.31 ng/mL for HYAL-1. If the measured concentration was below the detection limit, the DL value was assigned to the sample. The intra-assay coefficients of variation were 4.0 ± 2.9% for HA and 8.6 ± 7.9% for HYAL-1, respectively.

### Statistical analysis

Statistical analyses were performed with JASP 0.11.1 (Amsterdam, Netherlands) and Graph Pad Prism 5.0 (GraphPad Software, San Diego, CA, USA). The normality of the data was assessed with the Shapiro–Wilk test, which showed non-parametric distribution for HMW-HA and HYAL-1 levels. Clinical data, categorical variables and biomarkers were compared between OSA and control groups with t-test, Mann–Whitney U-test and Chi-square test. We applied non-parametric ANCOVA after adjustment on age, gender, BMI and smoking to evaluate the differences in HMW-HA and HYAL-1 levels as well as HMW-HA/HYAL-1 ratio between OSA and control groups as well as among controls, mild, moderate and severe patients with OSA. Comparisons between the OSA and control groups were performed also after excluding outlier data (defined as 75th percentile + 1.5 × interquartile range) as well as patients with chronic airway disease (asthma and COPD) and chronic heart failure. Further sensitivity analyses were performed when the comparisons were adjusted for statin and steroid use. Plasma HA and HYAL-1 levels were compared with clinical parameters and markers of sleep architecture with the Spearman test. The HMW-HA, HYAL-1 and HMW-HA/HYAL-1 results are presented as median with interquartile range. A p-value < 0.05 was considered significant.

The sample size was calculated to find difference in HMW-HA or HYAL-1 between the OSA and control groups with an effect size of 0.60, α error probability of 0.05 and power of 0.80 taking into the asymptotic relative efficiency of non-parametric tests. Post-hoc sensitivity analyses revealed that using this sample size we were able to find correlations with greater than − 0.19 or 0.19 critical r values^51^.

## Data Availability

The data are available from the corresponding author on request.
